# Comparative effectiveness and safety of tranexamic acid plus diluted epinephrine to control blood loss during total hip arthroplasty: a meta-analysis

**DOI:** 10.1186/s13018-018-0948-1

**Published:** 2018-09-21

**Authors:** Zhao Wang, Hao-jie Zhang

**Affiliations:** 1 Department of Orthopedics, Jingjiang People’s Hospital, Jingjiang, China; 2 Department of Orthopaedics, The 82rn Hospital of People’s Liberation Army of China, No. 100, Jiankangdong Road, Huai’an, Jiangsu China

**Keywords:** Tranexamic acid, Blood loss, Total hip arthroplasty, Meta-analysis

## Abstract

**Background:**

The standard protocol to achieve haemostasis during total hip arthroplasty (THA) is uncertain. Tranexamic acid plus diluted epinephrine (DEP) and tranexamic acid (TXA) alone are the two most common alternatives. The purpose of this study was to compare the efficacy and safety of TXA plus DEP to treat blood loss in THA patients.

**Methods:**

Published randomized controlled trials (RCTs) were identified from the following electronic databases: PubMed, Embase, Web of Science, Cochrane Library and Google from inception to July 10, 2018. Studies comparing TXA plus DEP with TXA alone to treat blood loss were included. Either a random-effects model or a fixed-effects model was used for meta-analysis depending on the heterogeneity. We used the need for transfusion as the primary outcome. Stata 12.0 was used for meta-analysis.

**Results:**

Six studies involving 703 patients were included in the present meta-analysis. The pooled results demonstrated that TXA plus DEP was associated with a lower transfusion rate than TXA alone (RR = 0.57, 95% CI 0.38–0.86, *P* = 0.006). Furthermore, TXA plus DEP was associated with less total blood loss and hidden blood loss by approximately 209.79 ml and 297.74 ml, respectively, than TXA alone. There was no significant difference in terms of intraoperative blood loss or the occurrence of deep venous thrombosis or haematoma between the TXA plus DEP and TXA alone groups (*P* > 0.05).

**Conclusions:**

Our meta-analysis suggested that TXA plus DEP significantly decreased the need for transfusion, total blood loss and hidden blood loss among THA patients. Furthermore, TXA plus DEP did not increase the occurrence of DVT or haemostasis. Additional long-term follow-up RCTs are needed to identify the optimal doses of TXA and DEP.

**Electronic supplementary material:**

The online version of this article (10.1186/s13018-018-0948-1) contains supplementary material, which is available to authorized users.

## Introduction

Total hip arthroplasty (THA) is an effective treatment for end-stage hip osteoarthritis (OA) [1]. By 2030, the demand for primary THA is estimated to increase to 572,000 [2]. THA is associated with a large amount of intraoperative blood loss and hidden blood loss [3]. Extensive blood loss results in cardiovascular complications and the need for a blood transfusion [4, 5]. Blood transfusion carries the risk of hepatitis virus transmission and immunomodulation, increasing economic costs and prolonging the length of hospital stay [6]. Therefore, there is an urgent need to identify a safe, effective method of reducing blood loss and blood transfusions after THA.

Several alternatives are available for minimizing blood loss after THA. These include topical fibrin sealants, topical or intravenous tranexamic acid (TXA) [7, 8], aminocaproic acid [3, 9] or diluted epinephrine (DEP) [10]. Recently, administration TXA plus DEP has become popular for THA patients [11]. DEP enhances coagulation by several mechanisms [12]. Nevertheless, whether TXA plus DEP is superior to TXA alone remains unclear. To further explore these issues and to identify the best haemostatic techniques for THA, we performed a meta-analysis of all the available randomized controlled trials (RCTs) of patients with THA.

## Methods

This review was conducted according to the Preferred Reporting Items for Systematic Reviews and Meta-Analysis Statement issued in 2011 [13]. Ethical approval was not necessary for this study, as only de-identified pooled data from individual studies were analysed.

### Search strategies

We searched PubMed, Embase and Cochrane CENTRAL for relevant studies from the time of inception of these databases to July 10, 2018. The following groups of keywords and medical terms were used for the literature search: “tranexamic acid” AND “epinephrine” (OR “total hip arthroplasty” OR “total hip replacement” OR arthroplasty OR “THA” “THR”) AND (random* OR prospective* OR trial*). The language was not restricted to English. We also conducted an additional search by screening the references of eligible studies.

### Study eligibility

We evaluated each identified RCT against the following predetermined selection criteria:

i. Study population: adults with hip OA eligible for primary THA.
ii. Interventions: the review focused on topical or intravenous TXA plus topical DEP, which are commonly used in the management of blood loss after THA, as commonly reported in the literature.
iii. Comparator: direct comparisons among any of the four core therapeutic interventions (i.e. DEP alone, topical or intravenous TXA alone and a control group).
iv. Outcome measures: the primary outcomes for this review were the need for transfusion, total blood loss, blood loss in drainage and the occurrence of deep venous thrombosis (DVT).

### Data extraction

Two authors independently extracted the general characteristics and outcomes from the included studies. The following data were extracted from each study: first author, publication year, location, age and number of patients in the intervention and control groups, doses of TXA and DEP, outcomes, transfusion threshold and follow-up. The differences in the extracted data were discussed by a panel of all the reviewers. When there were no clear data or missing data from the included studies, we tried to contact the corresponding author to obtain the relevant data.

### Quality assessment

Two reviewers independently evaluated the risk of bias using the Cochrane risk-of-bias tool. Seven major domains of bias (selection bias (random sequence generation), selection bias (allocation concealment), performance bias, detection bias, attrition bias, reporting bias and other bias) in each trial were reviewed. Disagreements between the reviewers were resolved by discussion.

### Statistically analysis

The risk ratios (RRs) with 95% confidence intervals (CIs) were calculated for the need for transfusion and the occurrence of DVT. The weighted mean difference (WMD) and corresponding CIs were calculated for continuous data (total blood loss, blood loss in drainage). Heterogeneity was explored for all the meta-analyses and quantified using *I*^2^ statistics. When *I*^2^ value was > 50%, this was considered substantial heterogeneity between studies. If there was a large clinical heterogeneity, a random-effects model was applied to pool the outcome data. A *P* value < 0.05 was considered statistically significant. All statistical analyses were performed using Stata 12.0 (Stata Corp., College Station, TX). Subgroup analysis was further performed according to the following variables: risk of bias (low or unclear/high), IV TXA dose (≥ 2 g or < 2 g), topical dose (≥ 2 g or < 2 g) and transfusion protocol (strict or loose). We categorized the TXA dose of 30 mg/kg into the subgroup of ≥ 2 g. Sensitivity analysis was also performed by omitting each of the studies in turn.

### Quality of evidence assessment

We used the Grading of Recommendations Assessment, Development and Evaluation (GRADE) methodology to assess the quality of evidence. The assessment includes five items: risk of bias, inconsistency, indirectness, imprecision and publication bias. Each outcome was rated as high, moderate, low or very low. Summary tables were constructed using GRADE Pro version 3.6 (GRADE Working Group).

## Results

### Search results

**Fig. 1:**
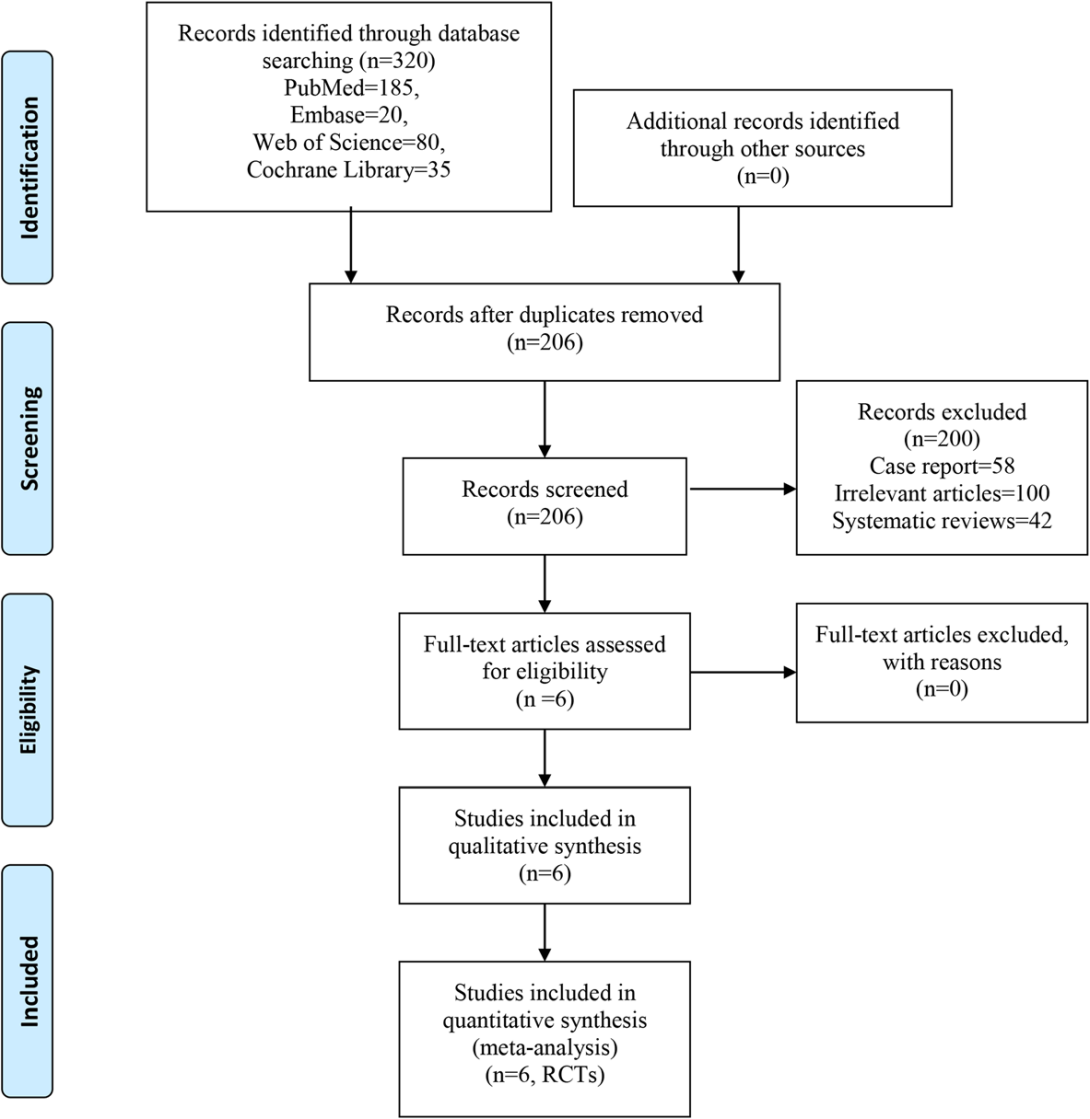
Flow diagram of the study selection process

A flowchart of study search and selection is presented in Fig. 1. We identified 320 references (PubMed = 185, Embase = 20, Web of Science = 80, Cochrane Library = 35) in our initial literature search. There were no additional records identified through other sources. After removing duplicates using Endnote X7 software, there were 206 studies remaining. Subsequently, 200 studies were excluded according to the inclusion criteria. Finally, 6 trials with 703 patients met our inclusion criteria and were included in the meta-analysis [14–19]. The general characteristics of the included studies can be seen in Table 1. All trials were published after the year 2015. Five studies were performed in China, and one was performed in Denmark. The mean age of the patients ranged from 50.0 to 69 years. Patients’ ages ranged from 21 to 65 years, and all were less than 100 years old.

**Table 1:** General characteristic of the included studies. 1, need for transfusion; 2, total blood loss; 3, hidden blood loss; 4, intraoperative blood loss; 5, the occurrence of DVT; 6, the occurrence of hematoma

Author	Country	Age (years, I/C)	No. of patients (*n*)	Interventions	Dose of intervention	Outcomes	Transfusion threshold	Follow-up
Gao F 2015	China	58.6 vs 61.7	53 vs 54	Topical TXA + topical DEP vs topical TXA	TXA (3 g), DEP (0.25 mg, 1:200000)	1, 2, 3, 4, 5, 6	Hb < 70 g/l	3 months
Jans O 2016	Denmark	67 vs 69	50 vs 50	Intravenous TXA + intravenous DEP vs intravenous TXA	TXA (1 g), DEP (0.05 μg kg^−1^ min^−1^)	2,3,	Hb < 80 g/l	At discharge
Liu JL 2018	China	50.0 vs 50.2 vs 51.8	65 vs 65 vs 65	Intravenous TXA + DEP vs intravenous TXA + topical DEP vs intravenous TXA	IV DEP (1 mg), IV TXA (10 mg/kg), topical DEP (0.25 mg, 1:200000)	1, 2, 3, 4, 5, 6	Hb < 70 g/l	2 weeks
Wang JW 2017	China	67 vs 69	45 vs 45	Topical TXA + topical DEP vs topical TXA	TXA (3 g), DEP (0.25 mg, 1:200000)	1, 2, 3, 4, 5, 6	Hb < 80 g/l	2 months
Zhang JK 2017	China	62.5 vs 63.1	21 vs 34	Topical TXA + topical DEP vs topical TXA	TXA (3 g), DEP (0.25 mg, 1:200000)	1, 2, 3, 4, 5, 6	Hb < 80 g/l	6 months
Zhang JZ 2017	China	59.8 vs 60.3 vs 58.6	52 vs 52 vs 52	Topical 1 g TXA + topical low dose DEP vs topical 1 g TXA + topical high dose DEP vs topical TXA	TXA (1 g), DEP (0.125 mg, 0.25 mg, 1:200000)	1, 2, 3, 4, 5	Hb < 70 g/l	3 months

### Quality assessment

**Fig. 2:**
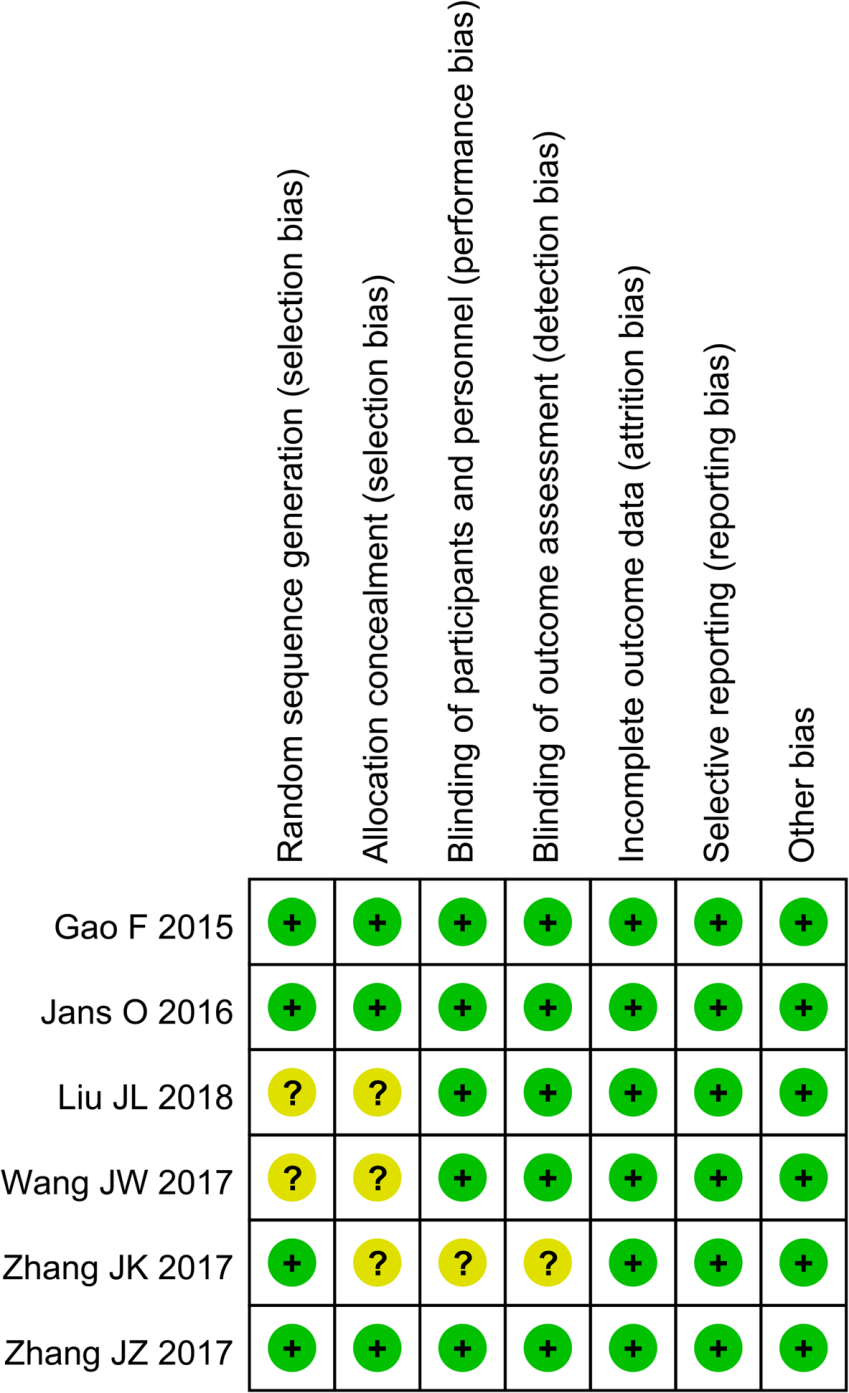
Risk of bias summary for the included RCTs. +, low risk of bias; −, high risk of bias; ?, unclear risk of bias

**Fig. 3:**
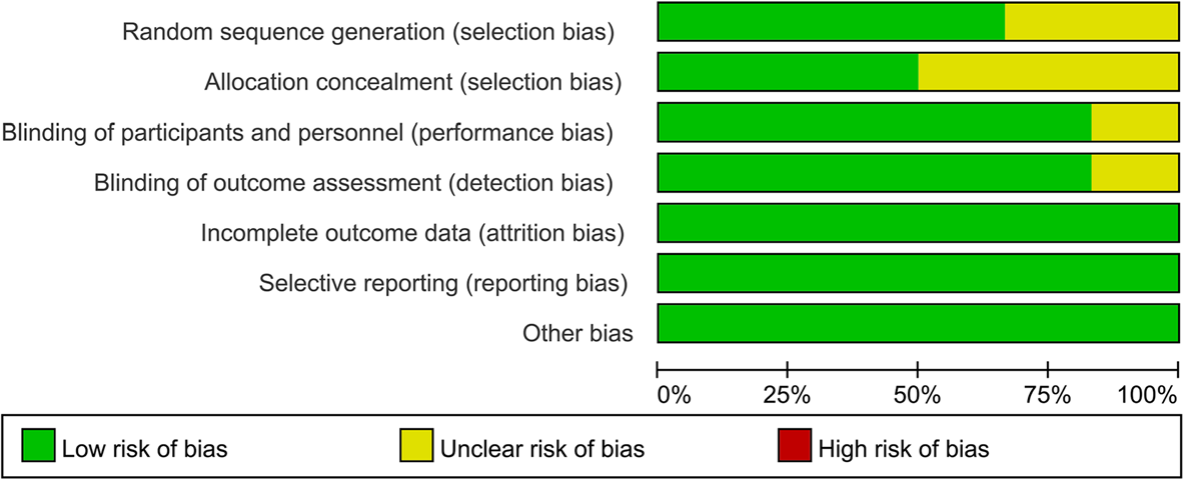
Risk of bias graph for the included RCTs

Data regarding the risk of bias summary and risk of bias graphs for each study are presented in Figs. 2 and 3, respectively. Three studies had a low risk of bias. The other studies were considered to have an unclear risk of bias.

### Quality of evidence assessment

The GRADE evidence profiles are presented in Additional file 1. The GRADE level of evidence was low for total blood loss, hidden blood loss and intraoperative blood loss; it was moderate for the need for transfusion and the occurrence of DVT and haematoma.

### Results of the meta-analysis

#### Need for transfusion

**Fig. 4:**
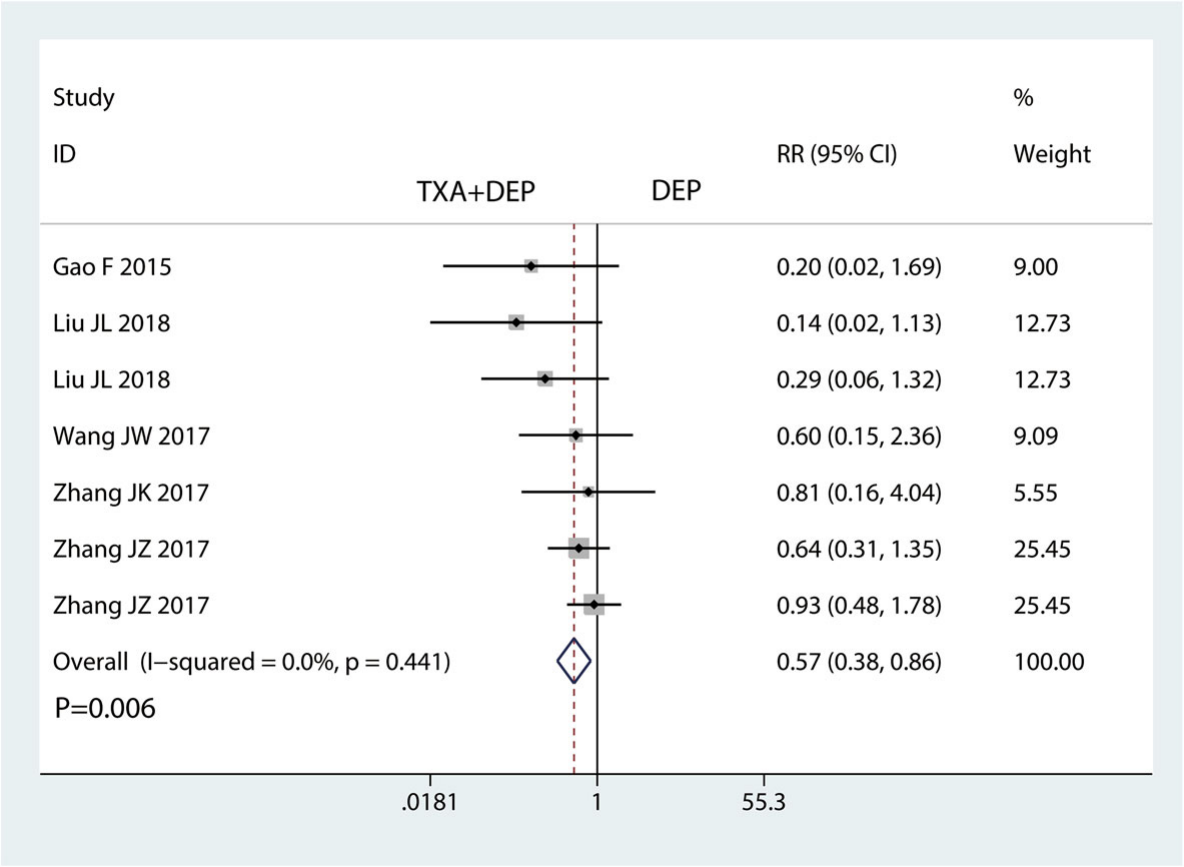
Forest plot for the comparison of the need for transfusion between the TXA plus DEP and TXA alone groups

Five studies were available with information regarding transfusion rate. The pooled results demonstrated that TXA plus DEP was associated with a lower transfusion rate than TXA alone (RR = 0.57, 95% CI 0.38–0.86, *P* = 0.006, Fig. 4). No heterogeneity was detected (*I*^2^ = 0%, *P* = 0.441), and thus, a fixed-effects model was used. The results of the subgroup analysis are shown in Table 2. The findings of a decreased need for transfusion were consistent for different doses of TXA except for the risk of bias and transfusion protocol.

**Table 2:** Subgroup analysis for the need for transfusion

Subgroup	No. trials	RR (95% CI)	*P* value	*I*^2^ (%)	Test of interaction, *P*
Risk of bias					
Low	4	0.60 (0.38, 0.95)	0.028	35.5	0.047
Unclear/high	3	0.50 (0.21, 1.16)	0.105	0	
Dose of TXA					
Low	4	0.60 (0.38, 0.93)	0.014	34.1	0.125
High	3	0.50 (0.16, 0.84)	0.023	0	
Transfusion protocol					
Strict	3	0.43 (0.18, 1.05)	0.064	0	0.043
Loose	4	0.63 (0.40, 0.98)	0.041	14.5	

#### Total blood loss

**Fig. 5:**
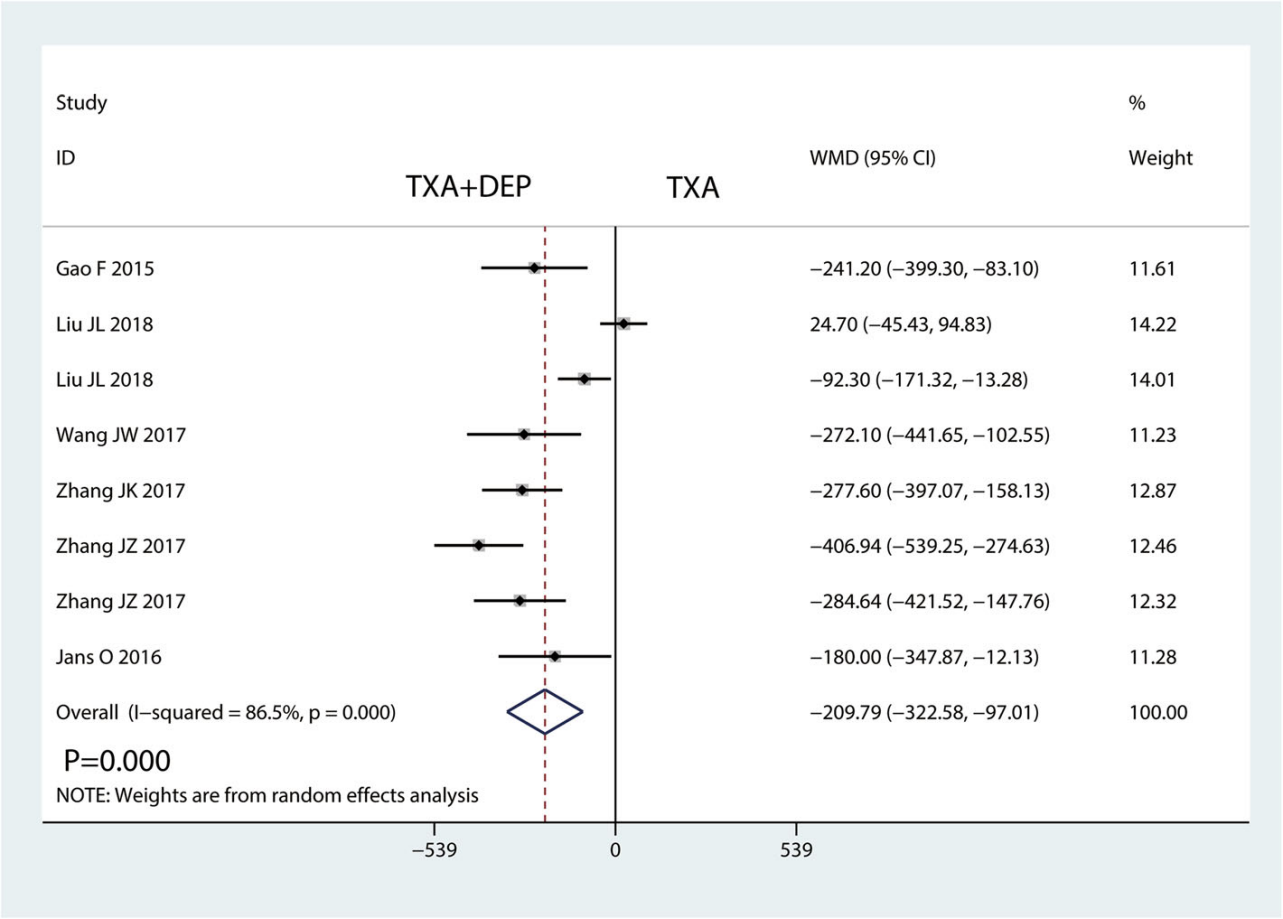
Forest plot for the comparison of total blood loss between the TXA plus DEP and TXA alone groups

Five studies were available for analysis of total blood loss. TXA plus DEP led to significantly less total blood loss than TXA alone (WMD = − 209.79, 95% CI − 322.58 to − 97.02, *P* = 0.000; *I*^2^ = 86.5%, *P* = 0.000, Fig. 5). Thus, we used a random-effects model to pool the relevant data.

#### Hidden blood loss

**Fig. 6:**
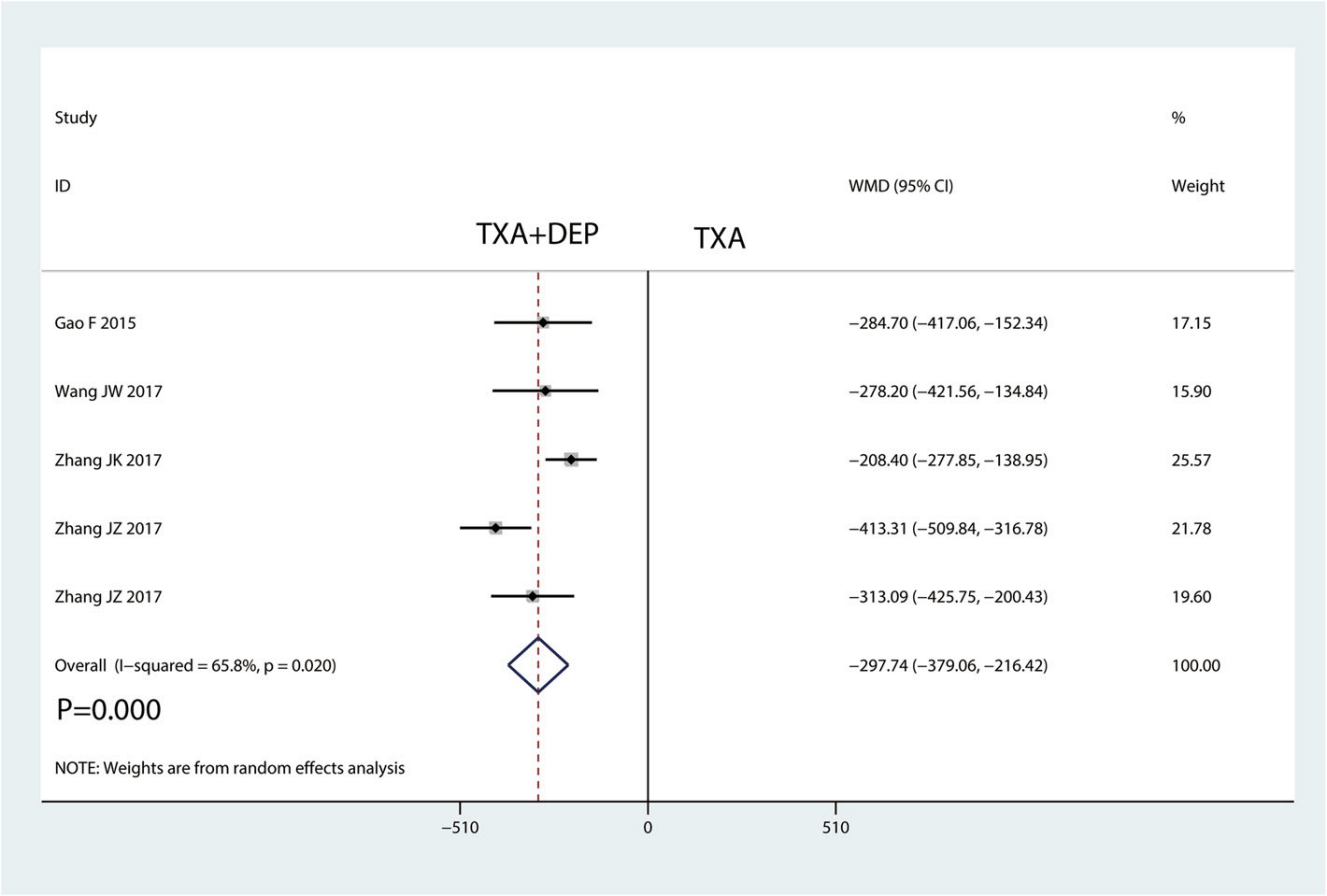
Forest plot for the comparison of hidden blood loss between the TXA plus DEP and TXA alone groups

Five studies were available for analysing hidden blood loss. TXA plus DEP led to significantly less hidden blood loss than TXA alone (WMD = − 297.74, 95% CI − 379.06 to − 216.42, *P* = 0.000; *I*^2^ = 65.8%, *P* = 0.020, Fig. 6). Thus, we used a random-effects model to pool the relevant data.

#### Intraoperative blood loss

**Fig. 7:**
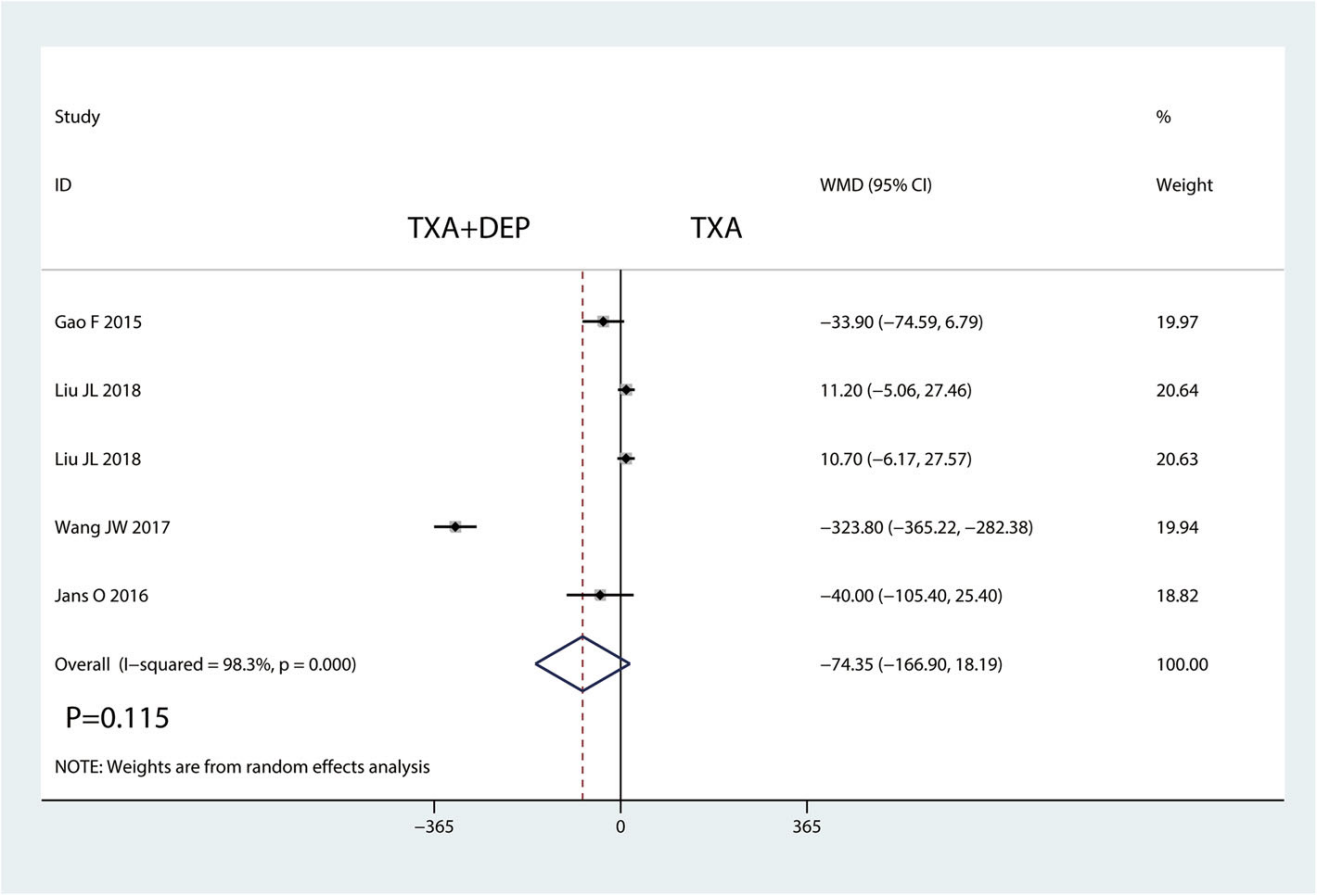
Forest plot for the comparison of intraoperative blood loss between the TXA plus DEP and TXA alone groups

Four studies were available for analysis of intraoperative blood loss. TXA plus DEP led to significantly less hidden blood loss than TXA alone (WMD = − 74.35, 95% CI − 166.90 to 18.19, *P* = 0.115; *I*^2^ = 98.3%, *P* = 0.000, Fig. 7). Thus, we used a random-effects model to pool the relevant data.

### The occurrence of DVT and haematoma

**Fig. 8:**
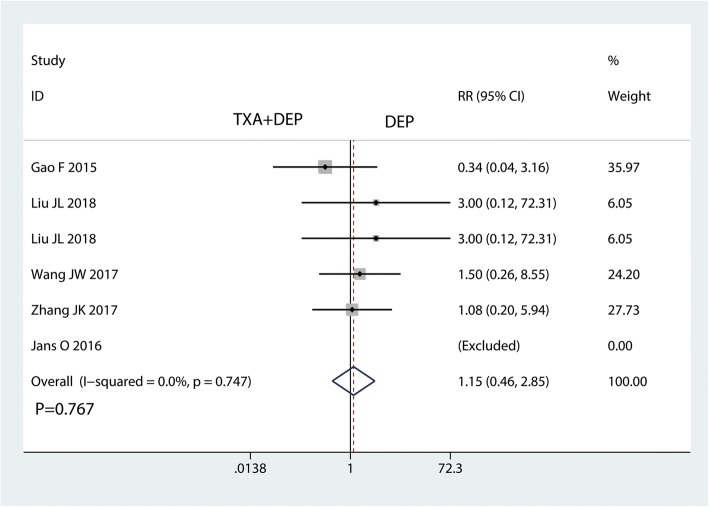
Forest plot for the comparison of the occurrence of DVT between the TXA plus DEP and TXA alone groups

**Fig. 9:**
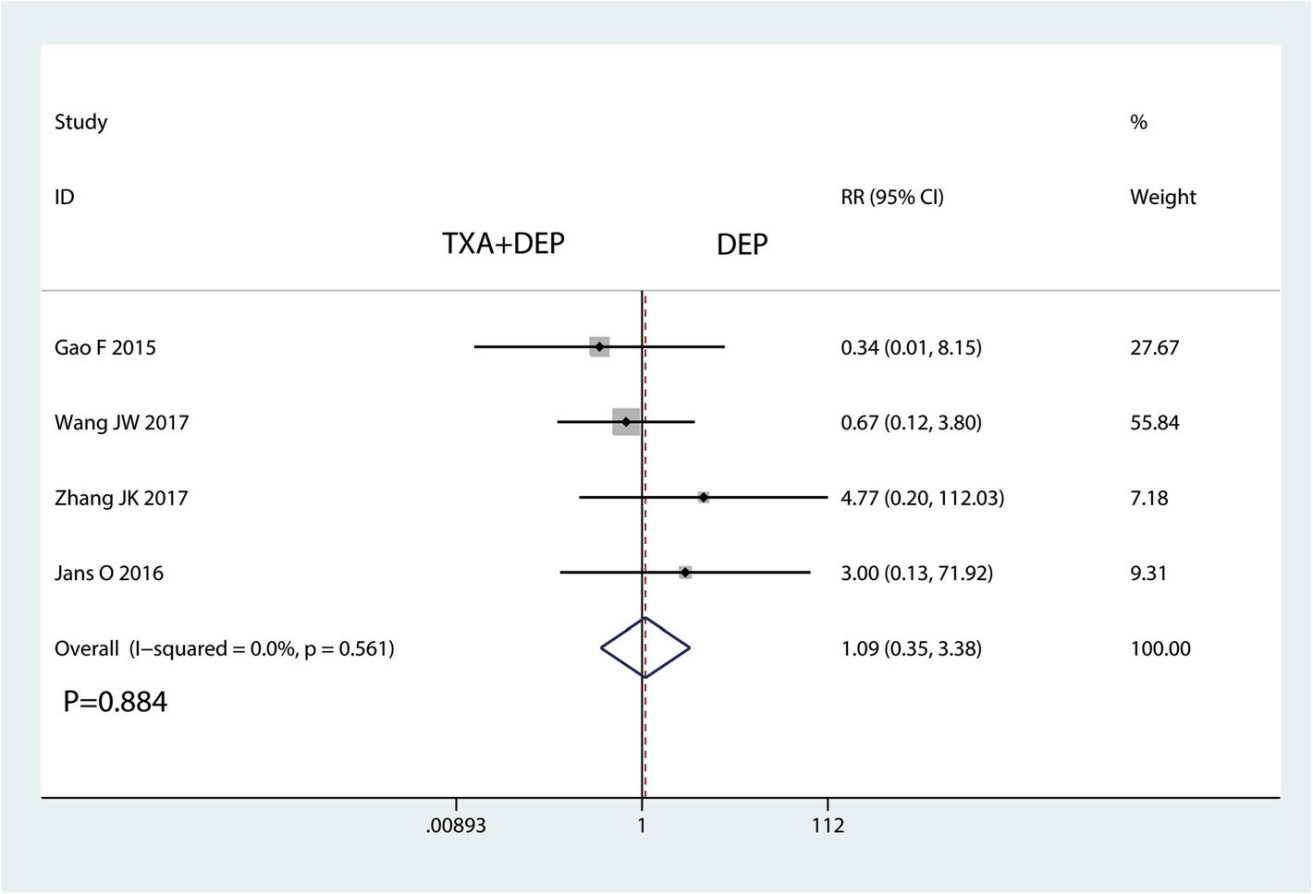
Forest plot for the comparison of the occurrence of haematoma between the TXA plus DEP and TXA alone groups

Five studies reported the occurrence of DVT. There was no significant difference in the occurrence of DVT between the TXA plus DEP and TXA alone groups (RR = 1.15, 95% CI 0.46–2.85, *P* = 0.767, Fig. 8). No heterogeneity was detected (*I*^2^ = 0%, *P* = 0.747); thus, a fixed-effects model was used. Four studies reported the occurrence of haematoma. There was no significant difference in the occurrence of DVT between the TXA plus DEP and the TXA alone groups in terms of the occurrence of haematoma (RR = 1.09, 95% CI 0.35–3.38, *P* = 0.884, Fig. 9). No heterogeneity was detected (*I*^2^ = 0%, *P* = 0.561); thus, a fixed-effects model was sued.

### Sensitivity analysis, publication bias

**Fig. 10:**
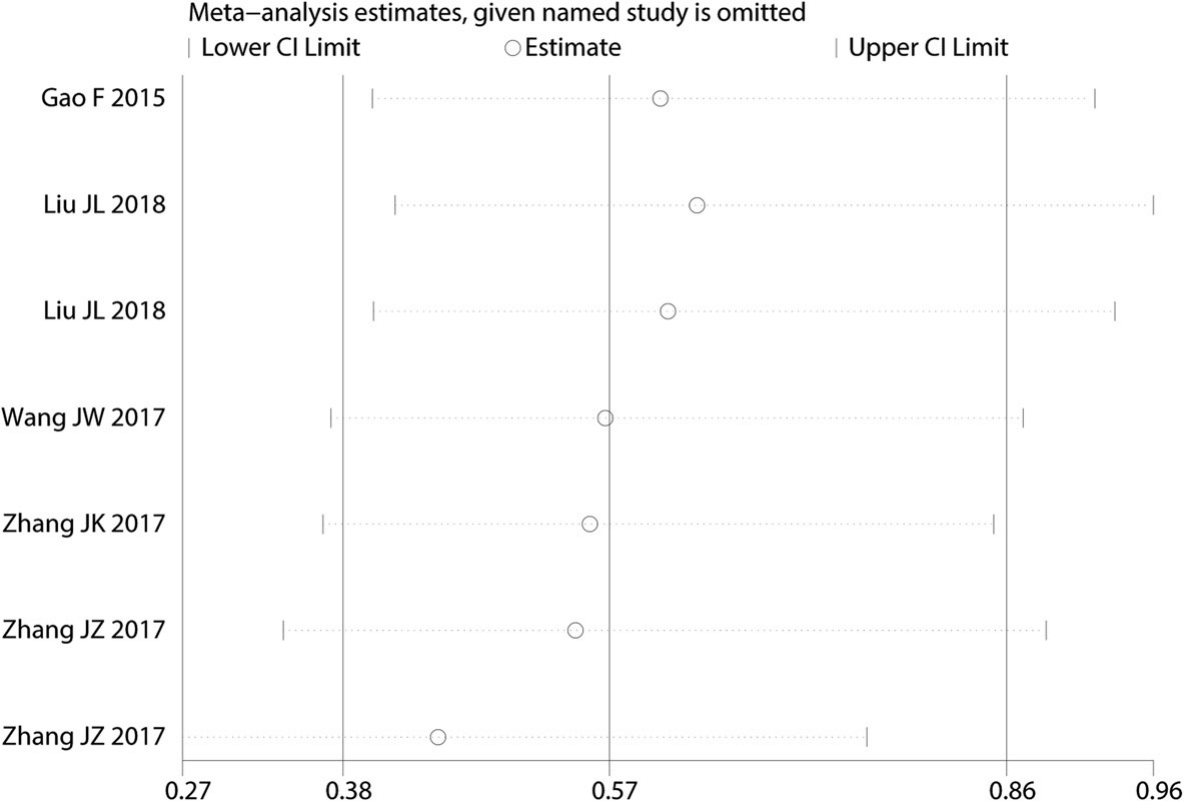
Sensitivity analysis for the need for transfusion after omitting each study in turn

**Fig. 11:**
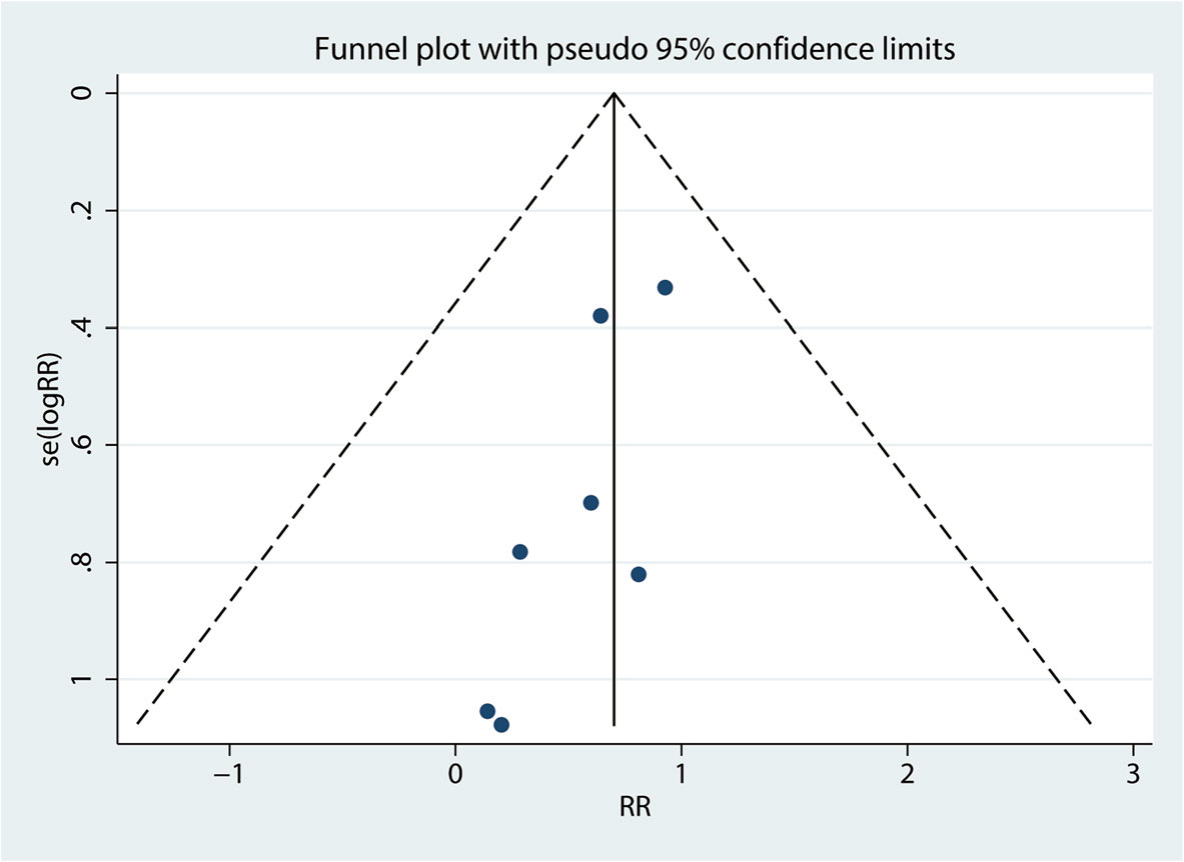
Funnel plot of the need for transfusion

**Fig. 12:**
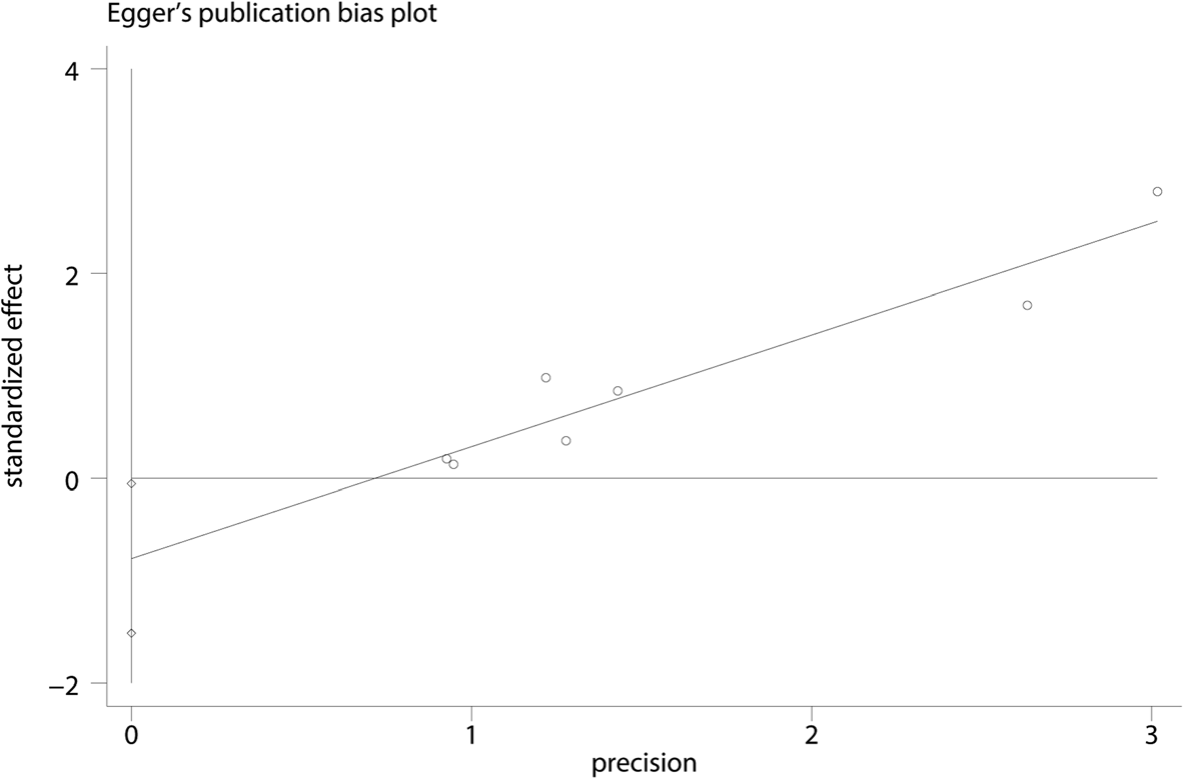
Egger’s test for publication bias for the need for transfusion

We performed a sensitivity analysis for the need for transfusion (Fig. 10. The results showed that after omitting the included studies, in turn, the overall effects did not change. The funnel plots were visually assessed and revealed no asymmetry (Fig. 11); no evidence of publication bias was determined by the Egger linear regression test for the need for transfusion (*P* = 0.72, Fig. 12),

## Discussion

In the current meta-analysis, we evaluated the efficacy and safety of TXA plus DEP for patients with THA. On the basis of the pooled estimates, TXA plus DEP was associated with significantly less total blood loss and subsequent need for transfusion than TXA alone. The use of tanezumab was not associated with a significantly increased risk of DVT or haematoma.

This was not the first meta-analysis. Yu et al. [20] conducted a meta-analysis comparing TXA plus DEP for blood loss after total joint arthroplasty (THA and total knee arthroplasty). Thus, we could not determine whether TXA plus DEP was certain to have a significant influence on controlling blood loss among patients undergoing THA alone. Moreover, Yu et al. [20] only included two studies that focused on THA. In this meta-analysis, we ultimately included six studies totalling 703 patients, adding the statistical power of at least 535 cases. Our meta-analysis was the latest and the most comprehensive one, and it generally concurs and further reinforces the results of the previous meta-analysis. Finally, we performed a subgroup-analysis and evaluated the quality of evidence using GRADE to help healthcare professionals make clinical decisions.

The current meta-analysis demonstrated that TXA plus DEP has a beneficial effect on total blood loss. TXA plus DEP was associated with less total blood loss by 209.79 ml than TXA alone. Several meta-analyses have found that TXA has a beneficial role in reducing blood loss in THA patients without increasing DVT occurrence [21, 22]. TXA can be administered by several routes including topical [23], intravenous [24] and oral [25]. Studies have shown that there was no significant difference among these routes in terms of the total blood loss. Concerning DEP, Jans O et al. [15] strongly suggested that intravenous DEP could be beneficial for reducing blood loss after THA. The administration of low-dose DEP could act as a procoagulant by increasing platelet aggregation, resulting in an instant 20–30% increase in platelet count [26]. Furthermore, DEP could activate α-adrenergic and β-adrenergic receptors; therefore, DEP could stimulate the release of several coagulation factors [27].

We measured hidden blood loss between the TXA plus DEP and TXA alone groups. We found that TXA plus DEP significantly reduced hidden blood loss by 297.74 ml compared with TXA alone. In THA patients, significant blood loss can occur after wound closure, and the proportion of this blood loss is called hidden blood loss. Hidden blood loss accounts for as much as 60% of the total perioperative blood loss [28]. With the administration of DEP, the procoagulant effects could last for 1–2 h; therefore, oozing could be decreased.

Regarding complications, we measured the occurrences of DVT and haematoma formation. We found that there was no significant difference between the occurrences of DVT and haematoma formation. Regarding the administration of TXA, DVT was the major concern. Several meta-analyses have identified that administration with TXA does not increase the occurrence of DVT [29]. Due to the incidence rate being relatively small, there is a need for studies to further clarify the risk [30].

There were several limitations in this meta-analysis: (1) the doses of TXA and DEP varied among the included studies, and the optimal doses of TXA and DEP require further exploration; (2) heterogeneity was large in terms of total blood loss and hidden blood loss, and these two outcomes should be interpreted cautiously; (3) the follow-up period varied among the included studies; thus, complications of TXA plus DEP may have been underestimated; and (4) the sample size was relatively small in the included studies; therefore, high-quality large-scale sample RCTs are needed.

## Conclusion

This meta-analysis suggests that TXA plus DEP has benefits in terms of total blood loss, hidden blood loss and the need for transfusion. Furthermore, TXA plus DEP had no influence on the occurrence of DVT or haematoma formation. Given all the shortcomings of this meta-analysis, further research and analysis are required to draw more reliable conclusions.

## Additional file

Additional file 1: Grade evidence for the outcomes. (DOCX 14 kb)

## Abbreviations

CIs: Confidence intervals
DEP: Diluted epinephrine
DVT: Deep venous thrombosis
OA: Osteoarthritis
RCTs: Randomized controlled trials
RR: Risk ratio
THA: Total hip arthroplasty
TXA: Tranexamic acid
WMD: Weighted mean difference
