# Supplementary material for: Comparative effectiveness and safety of tranexamic acid plus diluted epinephrine to control blood loss during total hip arthroplasty: a meta-analysis
Source: J Orthop Surg Res. 2018 Sep 21;13:242. doi: 10.1186/s13018-018-0948-1 (PMC6151011; doi:10.1186/s13018-018-0948-1)
Supplement: Supplementary file 1 — Grade evidence for the outcomes. (DOCX 14 kb) [file 13018_2018_948_MOESM1_ESM.docx]

|  | | | | | | |
| --- | --- | --- | --- | --- | --- | --- |
| **Patient or population:** patients with [health problem] **Settings:**  **Intervention:** outcomes | | | | | | |
| **Outcomes** | **Illustrative comparative risks* (95% CI)** | | **Relative effect (95% CI)** | **No of Participants (studies)** | **Quality of the evidence (GRADE)** | **Comments** |
|  | Assumed risk | Corresponding risk |  |  |  |  |
|  | **Control** | **Outcomes** |  |  |  |  |
| **need for transfusion** | **Study population** | | **RR 0.57**  (0.38 to 0.86) | 720 (7 studies) | ⊕⊕⊕⊝ **moderate** |  |
|  | **153 per 1000** | **87 per 1000** (58 to 131) |  |  |  |  |
|  | **Moderate** | |  |  |  |  |
|  | **111 per 1000** | **63 per 1000** (42 to 95) |  |  |  |  |
| **total blood loss** |  | The mean total blood loss in the intervention groups was **214.25 lower** (339.08 to 89.41 lower) |  | 720 (7 studies) | ⊕⊕⊝⊝ **low** |  |
| **hidden blood loss** |  | The mean hidden blood loss in the intervention groups was **297.74 lower** (379.06 to 216.42 lower) |  | 460 (5 studies) | ⊕⊕⊝⊝ **low** |  |
| **intraoperative blood loss** |  | The mean intraoperative blood loss in the intervention groups was **74.35 lower** (166.9 lower to 18.19 higher) |  | 557 (5 studies) | ⊕⊕⊝⊝ **low**^1^ |  |
| **the occurrence of DVT** | **Study population** | | **RR 1.15**  (0.46 to 2.85) | 612 (6 studies) | ⊕⊕⊕⊝ **moderate** |  |
|  | **26 per 1000** | **29 per 1000** (12 to 73) |  |  |  |  |
|  | **Moderate** | |  |  |  |  |
|  | **22 per 1000** | **25 per 1000** (10 to 63) |  |  |  |  |
| **the occurrence of hematoma** | **Study population** | | **RR 1.09**  (0.35 to 3.38) | 352 (4 studies) | ⊕⊕⊕⊝ **moderate** |  |
|  | **22 per 1000** | **24 per 1000** (8 to 74) |  |  |  |  |
|  | **Moderate** | |  |  |  |  |
|  | **9 per 1000** | **10 per 1000** (3 to 30) |  |  |  |  |
| *The basis for the **assumed risk** (e.g. the median control group risk across studies) is provided in footnotes. The **corresponding risk** (and its 95% confidence interval) is based on the assumed risk in the comparison group and the **relative effect** of the intervention (and its 95% CI).  **CI:** Confidence interval; **RR:** Risk ratio; | | | | | | |
| GRADE Working Group grades of evidence **High quality:** Further research is very unlikely to change our confidence in the estimate of effect.  **Moderate quality:** Further research is likely to have an important impact on our confidence in the estimate of effect and may change the estimate. **Low quality:** Further research is very likely to have an important impact on our confidence in the estimate of effect and is likely to change the estimate. **Very low quality:** We are very uncertain about the estimate. | | | | | | |
| ^1^ No explanation was provided | | | | | | |

**Supplement S1 Grade evidence for the outcomes.**
